# Air‐Stable 2D Intrinsic Ferromagnetic Ta_3_FeS_6_ with Four Months Durability

**DOI:** 10.1002/advs.202001722

**Published:** 2020-10-13

**Authors:** Jianwei Su, Mingshan Wang, Guiheng Liu, Huiqiao Li, Junbo Han, Tianyou Zhai

**Affiliations:** ^1^ State Key Laboratory of Material Processing and Die and Mould Technology School of Materials Science and Engineering Huazhong University of Science and Technology (HUST) Wuhan 430074 P. R. China; ^2^ Wuhan National High Magnetic Field Center and Department of Physics Huazhong University of Science and Technology (HUST) Wuhan 430074 P. R. China

**Keywords:** 2D materials, air‐stable 2D ferromagnetism, ferromagnetism, MOKE, Ta_3_FeS_6_

## Abstract

2D ferromagnetic materials provide an important platform for the fundamental magnetic research at atomic‐layer thickness which has great prospects for next‐generation spintronic devices. However, the currently discovered 2D ferromagnetic materials (such as, CrI_3_, Cr_2_Ge_2_Te_6_, and Fe_3_GeTe_2_) suffer from poor air stability, which hinders their practical application. Herein, intrinsic long‐range ferromagnetic order in 2D Ta_3_FeS_6_ is reported, which exhibits ultrahigh stability under the atmospheric environment. The intrinsic ferromagnetism of few‐layer Ta_3_FeS_6_ is revealed by polar magneto‐optical Kerr effect measurement, which exhibits giant MOKE response and has Curie temperature of ≈80 K. More importantly, few‐layer Ta_3_FeS_6_ nanosheet exhibits excellent air stability and its ferromagnetism remains unchanged after 4 months of aging under the atmosphere. This work enriches the family of 2D ferromagnetic materials, which will facilitate the research progress of spintronics.

## Graphical abstract

Ultrastable intrinsic ferromagnetism in two‐dimensional Ta_3_FeS_6_ is demonstrated. The Ta_3_FeS_6_ nanosheet exhibits giant magneto‐optical Kerr effect response of 48 mrad and ferromagnetic transition temperature (T_C_) of ≈80 K. Even more important, Ta_3_FeS_6_ exhibits excellent air stability and its ferromagnetism remains unchanged after 4 months of aging in air, indicating its potential application in spintronic devices under atmospheric environment.

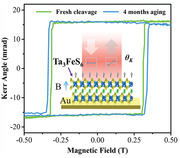

1

2D materials have generated great research interest due to their atomic flat interface structure, unique electronic structure (semiconducting, metallic, and superconducting), and a wide range of applications (high‐mobility transistors, ultra‐sensitive photodetectors, high‐efficiency energy conversion).^[^ ^1^, ^2^, ^3^, ^4^, ^5^, ^6^ ^]^ The emerging 2D ferromagnetic materials combining spin with the unique electronic structure of 2D materials exhibit novel magneto‐electric and magneto‐optical properties, which prefigures the rising of spintronics.^[^ ^7^, ^8^ ^]^ Recently, 2D ferromagnetism was discovered in chromium trihalides (CrX_3_, X = Cl, Br, I),^[^ ^9^, ^10^, ^11^, ^12^, ^13^ ^]^ Cr_2_Ge_2_Te_6_,^[^ ^8^, ^14^ ^]^ and Fe_3_GeTe_2_.^[^ ^15^, ^16^ ^]^ Magnetic CrI_3_ exhibits electrical‐field tunable 2D magnetism,^[^ ^17^, ^18^ ^]^ large interlayer tunneling magnetoresistance,^[^ ^13^, ^19^, ^20^ ^]^ and helical luminescence properties.^[^ ^21^ ^]^ However, most of the found 2D magnetic materials have poor stability in the atmospheric environment, i.e., CrI_3_ nanosheet degrades in the air in 15 min and the ferromagnetism of Fe_3_GeTe_2_ nanosheet vanishes under the atmosphere for a few hours, which hinders the scientific research of intrinsic 2D ferromagnetism and the practical applications.^[^ ^15^, ^20^ ^]^ Therefore, it is significant to explore high air‐stability 2D ferromagnetic materials for the research of 2D intrinsic ferromagnetism and the potential application of spintronics.

In this study, 2D intrinsic ferromagnetism in Ta_3_FeS_6_ was demonstrated with excellent air stability and strong magnetocrystalline anisotropy, which would be an ideal platform for investigating the 2D magnetism and spin devices. 2D ferromagnetic Ta_3_FeS_6_ nanosheets with different thickness were successfully prepared by the combination of crystal growth technique and Au‐assisted mechanical exfoliation strategy. Polar MOKE measurement demonstrated the robust 2D ferromagnetism of Ta_3_FeS_6_ with a giant MOKE rotation angle of 48 mrad. Further, temperature‐dependent MOKE measurement demonstrated that the Curie temperature of Ta_3_FeS_6_ nanosheet was 80 K. What's more, Ta_3_FeS_6_ nanosheet had excellent air stability and its ferromagnetism remained unchanged after 4 months aging. The air‐stable 2D ferromagnetism of Ta_3_FeS_6_ is an ideal platform for the investigation of spin devices and the construction of van der Waals magnetic heterostructures.

2D Ta_3_FeS_6_ was isolated from high‐crystalline single crystal via mechanical exfoliation. The Ta_3_FeS_6_ single crystal was grown by the chemical vapor transport (CVT) method, as illustrated in **Figure** **1**a (see the Experimental Section). Ta_3_FeS_6_ belongs to the space group of P6_3_22 (*No*. 182) and has hexagonal layered lattice structure with noncentrosymmetric and chiral feature, which is alternately stacked by Fe atoms and H‐phase TaS_2_ layers, as illustrated in Figure 1b.^[^ ^22^ ^]^ The crystal can also be denoted as Fe_1/3_TaS_2_ due to the $\sqrt{3}a\times \sqrt{3}a$ Fe atoms superlattice.^[^ ^23^ ^]^ The interlayer spacing of neighboring TaS_2_ is 0.614 nm and is larger than that of 2H‐TaS_2_ (0.605 nm) because of the intercalation induced lattice expansion.^[^ ^24^ ^]^ Besides, the Fe atoms in the adjacent layers have a complementary occupation and constitute honeycomb Fe lattice along the c axis of Ta_3_FeS_6_ crystal (Figure 1c).^[^ ^23^ ^]^ X‐ray diffraction (XRD) was employed to confirm the phase structure of Ta_3_FeS_6_ crystal. As shown in Figure 1d, the XRD pattern exhibited a series of strong sharp diffraction peaks which corresponded well with the [00l] planes of Ta_3_FeS_6_ crystal (JCPDS *No*. 22‐0360) and further demonstrated its layered features with the high‐crystalline quality.

**Figure 1:**
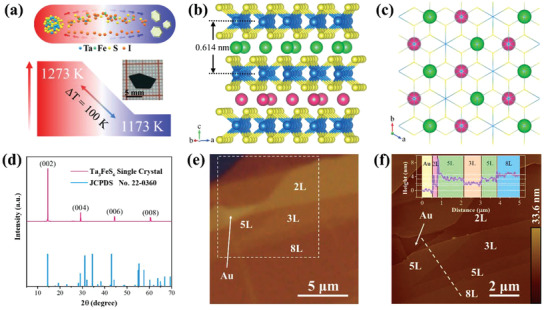
a) Schematic illustration of the CVT synthesis process; The top part of Figure a illustrates the material diffusion and reaction in a two‐end furnace; The below part shows the temperature gradient and optical image of as‐grown Ta_3_FeS_6_ single crystal. b) Crystal structure of Ta_3_FeS_6_ and the Fe atoms are indicated with different colors (top layer Fe atoms: green; under layer Fe atoms: red.) at the neighboring layers. c) Fe atoms arrangement viewed along the c axis. d) XRD pattern of Ta_3_FeS_6_ single crystal. e) OM image of Ta_3_FeS_6_ nanosheet on the Au substrate. f) AFM image of Ta_3_FeS_6_ nanosheet acquired at the square mark in Figure e. The inset figure shows the AFM height profile along with the white dashed line in figure f.

However, due to the ionic bonding effect between the Fe atoms and the TaS_2_ layers, the isolating of Ta_3_FeS_6_ nanosheets in experiment is still a great challenge.^[^ ^25^ ^]^ In recent years, metal‐assistant isolation technique was developed and had been proved to be efficient for large‐area preparation of various layered materials due to the chemically enhanced adhesion force between evaporated metal film and surface of materials.^[^ ^26^, ^27^ ^]^ Hence, the Au‐assisted mechanical exfoliation method was introduced to overcome the strong interlayer interaction and prepare atomic‐thin Ta_3_FeS_6_ nanosheet. The exfoliation process was illustrated in the Experimental Section and Figure S1 (Supporting Information). Figure 1e displays an optical microscope (OM) image of as‐exfoliated Ta_3_FeS_6_ nanosheet on 200 nm Au film and the few‐layer regions can be resolved by the optical contrast. The topography characterization was performed by an atomic force microscope (AFM) to identify the thickness of the Ta_3_FeS_6_ nanosheet. AFM image demonstrated that the cleaved nanosheet had bi‐ to eight‐layer thickness with distinct layer steps of ≈0.6 nm matching well with the interlayer distance of Ta_3_FeS_6_ crystal.^[^ ^24^ ^]^

To investigate the crystal structure and crystallization quality of the as‐prepared Ta_3_FeS_6_ nanosheet, transmission electron microscope (TEM) measurement was performed on an exfoliated nanosheet. **Figure** **2**a shows a TEM image of Ta_3_FeS_6_ nanosheet on a carbon grid and the transparent feature indicated its ultrathin character. High‐resolution TEM (HRTEM) image (Figure 2b) revealed the clear crystal lattices with a spacing of 0.28 nm, which corresponded to the (110) planes of hexagonal‐phase Ta_3_FeS_6_.^[^ ^28^ ^]^ To verify the elemental composition of the Ta_3_FeS_6_ crystal, the energy dispersive spectroscopy (EDS) analysis was carried out. The result showed that the as‐synthesized crystal contains Ta, Fe, and S elements with the ratio of approximate 3:1:6, as shown in Figure 2c. Furthermore, the selected area electron diffraction (SAED) was performed to acquire direct evidence of superlattice in Ta_3_FeS_6_, as shown in Figure 2d. In the SAED pattern, a set of hexagonal bright diffraction spots can be donated as (110) planes, which was attributed to the diffraction of the TaS_2_ frame.^[^ ^29^ ^]^ More intriguing, another concentric set of hexagonal spots was observed at (1/3 1/3 0) reciprocal positions, resulting from an ordered Fe superlattice of Ta_3_FeS_6_.^[^ ^23^ ^]^ All of the analysis above agreed well with the structural feature of Ta_3_FeS_6_ crystal. Additionally, the phase homogeneity of the Ta_3_FeS_6_ nanosheet was revealed by SAED. The SAED patterns (Figure 2d–f) acquired at different regions in the Ta_3_FeS_6_ nanosheet (Figure 2a) had identical diffraction spots and orientation (angle deviation smaller within 0.32°) confirming the structural homogeneity of Ta_3_FeS_6_ nanosheet.

**Figure 2:**
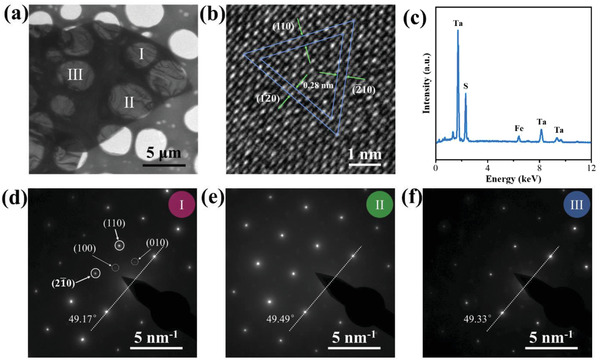
a) Low‐magnification TEM image of exfoliated Ta_3_FeS_6_ nanosheet. b) HRTEM image of exfoliated Ta_3_FeS_6_ nanosheet. c) EDS elemental analysis of the Ta_3_FeS_6_ sample. d–f) SAED pattern of the Ta_3_FeS_6_ sample acquired at different positions (shown in Figure 2a, I, II, and III). The $\sqrt{3}a\times \sqrt{3}a$ superlattice diffraction spots can be identified among the main spots.

Raman spectroscopy was further employed to investigate the spin dependence of Raman scattering of Ta_3_FeS_6_, since Raman spectra are structural‐sensitive and suitable for the research of the ferromagnetic‐paramagnetic phase transition.^[^ ^30^, ^31^, ^32^ ^]^ **Figure** **3**a shows the Raman spectra of Ta_3_FeS_6_ at 80 and 300 K. There were five Raman modes in the spectra which were labeled as P_1_, P_2_, … P_5_ with the frequency increasing. P_1_ and P_2_ peaks may attribute to the Fe superlattice layers and Fe‐Ta interaction.^[^ ^33^ ^]^ And P_3_, P_4_, and P_5_ can be identified as the Raman vibration mode of the H‐phase TaS_2_ layers, where P_3_ was ascribed to the two‐phonon signal, P_4_ was the longitudinal optical mode (E_2g_ mode), and P_5_ was the transverse optical vibration mode (A_1g_ mode).^[^ ^34^ ^]^ The Raman spectra at 80 and 300 K maintained a constant shape indicating that no crystal structure transition occured during the temperature‐changing process. To visually present the evolution of temperature‐dependent Raman spectra, the 2D contour map ranging from 80 K to 300 K was plotted, as shown in Figure 3b. As the temperature increases, P_2_, P_4_, and P_5_ exhibited obvious red‐shift (soften behavior).^[^ ^35^ ^]^ To analysis the soften behavior quantitatively, the relative peak position offset (*ie*. wavenumber change relative to room temperature, *ω*_T_ −*ω*_300 K_) were extracted and shown in Figure 3c; and Figure S2 (Supporting Information). P_5_ peak showed linear behavior along with temperature which can be fitted by the Lorentzian functions, as shown in Equation (1)

(1) $$\omega \left(T\right)=\omega_{0}+\chi T$$

where *ω*_0_ is the Raman peak position at 0 K, *χ* is the first‐order temperature coefficient of Raman vibration mode, and *T* is the Kelvin temperature.^[^ ^36^ ^]^ The temperature‐dependent Raman offset includes two components: the anharmonicities of the phonon modes which is temperature‐contribution dominant and the thermal expansion of the crystal which is due to the volume change, i.e., *χ* = *χ*_*T*_ + *χ*_*V*_.^[^ ^37^ ^]^ The fitted *χ* value of P_5_ was −(1.8 ± 0.1) × 10^−2^ cm^−1^ K^−1^, which was close to the reported first‐order temperature coefficient of other TMDs, such as MoS_2_ (*A*_1g_ mode, −0.016 cm^−1^ K^−1^) and WS_2_ (*A*_1*g*_ mode, −0.015 cm^−1^ K^−1^).^[^ ^37^, ^38^ ^]^ As demonstrated in previous works, the first‐order temperature coefficient is relative to the bonding‐type and structural configuration of materials.^[^ ^39^ ^]^ The similar *χ* value compared with MoS_2_ and WS_2_ indicated the layered nature in Ta_3_FeS_6_ crystal.

**Figure 3:**
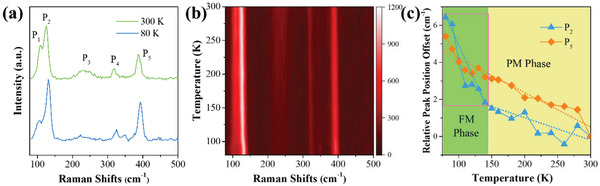
a) Raman spectra of Ta_3_FeS_6_ single crystal acquired at 80 and 300 K. b) 2D contour map of Raman spectra ranging from 80 K to 300 K. c) Relative Raman peak position offset‐temperature curves of P_2_ and P_5_. FM: ferromagnetic phase; PM: paramagnetic phase.

More intriguing, a turning point of P_2_ peak offset occured at around 145 K, as indicated in Figure 3c. The fitted first‐order temperature coefficient *χ* of the low‐temperature region (80–145 K) was −(8.7 ± 1.7) × 10^−2^ cm^−1^ K^−1^, which was 7.9 times larger than that at high‐temperature region (−(1.1 ± 0.2) × 10^−2^ cm^−1^ K^−1^, 145–300 K). Previous reports indicate that some of Raman peak positions or intensities change with decreasing temperature through the Curie temperature.^[^ ^40^, ^41^ ^]^ The prominent difference of Raman spectra between paramagnetic phase and ferromagnetic (or antiferromagnetic) phase can be summarized as follows: 1) New Raman modes due to the magnetic ordering (two‐magnon scattering); 2) Quenching of quasi‐elastic scattering from magnetic fluctuations; 3) Magnetic ordering induced Raman peak offset (frequency difference).^[^ ^30^, ^41^, ^42^ ^]^ Since the Curie temperature of Ta_3_FeS_6_ was ≈145 K, it can be deduced that the abnormal Raman offset was attributed to the ferromagnetic‐paramagnetic phase transition of Ta_3_FeS_6_, which was also observed in NiPS_3_.^[^ ^28^, ^41^ ^]^ Thus from this perspective, the P_2_ Raman peak can be used as an indicator of the magnetic ordering for Ta_3_FeS_6_.

Polar MOKE measurements have the advantage of high sensitivity, high spatial resolution, and non‐invasive detection, which are propitious to the research of 2D magnetic materials.^[^ ^43^ ^]^ **Figure** **4**a shows the schematic of the polar MOKE measurement of 2D Ta_3_FeS_6_. To obtain the out‐of‐plane ferromagnetism, a normal‐irradiated laser was used to detect the MOKE signals of samples.^[^ ^12^ ^]^ The schematic for the optical setup of polar MOKE is illustrated in Figure S3 (Supporting Information). Figure 4b shows Kerr rotation *θ*_K_ as a function of the magnetic field at different laser wavelengths for the Ta_3_FeS_6_ nanosheet (9 layer, Figure S4, Supporting Information). All the experiments were measured at 10 K with an incident light power of 3 µW. To rule out the possible contribution of Kerr signal by Au residues, the MOKE measurement was performed on bare Au film under the same condition and no obvious MOKE signal was observed (Figure S5, Supporting Information).^[^ ^44^, ^45^, ^46^ ^]^ Significantly, the magnetic hysteresis loop with a sharp switching edge demonstrated that the Ta_3_FeS_6_ nanosheet was ferromagnetic ordering with strong out‐of‐plane spin polarization.^[^ ^47^ ^]^ Previous theoretical calculation demonstrates that the local moments at Fe atoms induces a magnetic polarization of the Ta $5d_{Z^{2}}$ band, and the direction of polarization was parallel arranged along the c axis in the Ta_3_FeS_6_ structure, which agreed well with our experiment result.^[^ ^48^ ^]^ Furthermore, the MOKE signals displayed obvious dependence with the incident wavelength that some signals showed positive and others showed negative. The values of Kerr rotation *θ*_K_ and coercive field *H*_C_ were extracted as functions of wavelength, as shown in Figure 4c. The coercive field remains unchanged at different incident wavelengths, which was related to the intrinsic ferromagnetism of Ta_3_FeS_6_. However, the Kerr rotation *θ*_K_ exhibited strong wavelength dependence and changed its sign at ≈540 nm. The Kerr signal is proportional to the difference of spin‐up and spin‐down transition rate, which equals to joint density of states (JDOS).^[^ ^49^ ^]^ It is worth noting that the difference of JDOS between the spin‐up and spin‐down bands has photon energy dependence.^[^ ^50^ ^]^ When JDOS of the spin‐up band is larger than that of the spin‐down band, the Kerr signal shows positive and vice versa. Hence, the sign of Kerr signal changes at ≈540 nm may attribute to the sign change of the difference of JDOS.^[^ ^48^, ^49^ ^]^

**Figure 4:**
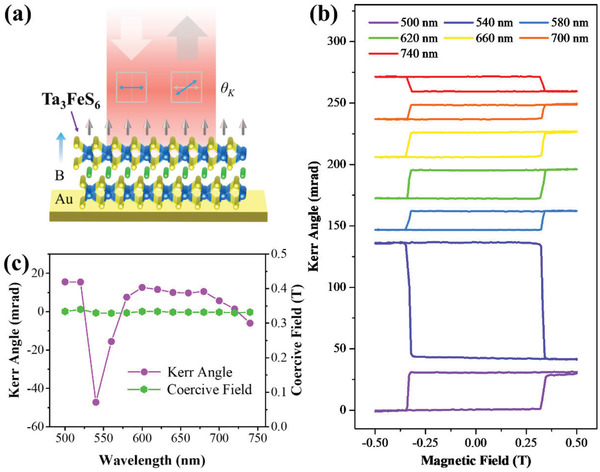
a) Schematic of the polar MOKE measurement of Ta_3_FeS_6_ nanosheet. b) Wavelength‐dependent polar MOKE signals of Ta_3_FeS_6_ nanosheet. c) Extractive Kerr angle and coercive field at different excitation wavelengths.

Temperature‐dependent polar MOKE measurements were performed to determine the Curie temperature of the Ta_3_FeS_6_ nanosheet. **Figure** **5**a shows Kerr rotation *θ*_K_ as a function of the magnetic field at several temperatures. With the increase of temperature, the hysteresis loop finally shrank away, indicating that the ferromagnetic phase was transforming into the paramagnetic phase. Figure 5b displays the Kerr rotation *θ*_K_ and the coercive field from Figure 5a. At 80 K, the coercive field of Ta_3_FeS_6_ nanosheet was zero, which implied that the Curie temperature T_C_ was around 80 K. Compared to the bulk sample (Figure S6, Supporting Information), the suppressed ferromagnetism of Ta_3_FeS_6_ nanosheet was due to the thermal fluctuations in the 2D system.^[^ ^8^ ^]^ Layer‐dependent ferromagnetism of Ta_3_FeS_6_ has been carried out, but it was hard to figure out the layer‐dependent phase diagram owing to the inhomogeneity of Fe atoms in the Ta_3_FeS_6_ crystal. The Fe content of Ta_3_FeS_6_ changed after the mechanical exfoliation process, as demonstrated by the EDS analysis (Figure S7, Supporting Information). Besides, the Curie temperature was sensitive to the Fe content in Ta_3_FeS_6_ crystal (Figure S8, Supporting Information). When there were more Fe vacancies in the superlattice, the Curie temperature of Ta_3_FeS_6_ crystal increased from 80 K to 145 K (Figure S9, Supporting Information).^[^ ^23^, ^47^ ^]^ The Fe vacancies arose from the crystal growth process. During the crystal growth process, the Fe atoms diffused to the TaS_2_ frame. Affected by the growth condition, such as the non‐homogeneous temperature distribution, the inhomogeneous diffusion of Fe atoms resulted in the fluctuation of Fe concentration in different layers of Ta_3_FeS_6_ crystal.

**Figure 5:**
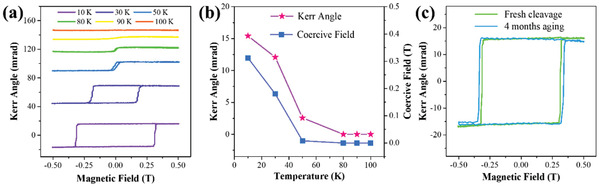
a) Temperature‐dependent polar MOKE measurement of Ta_3_FeS_6_ nanosheet. b) Extractive Kerr angle and coercive field at different temperatures. c) MOKE signal of Ta_3_FeS_6_ nanosheet acquired before and after 4 months aging under atmospheric conditions.

A significant obstacle for the research and application of 2D ferromagnetic materials is their air stability.^[^ ^8^, ^12^, ^15^ ^]^ Here, aging measurement was performed for researching the stability of 2D Ta_3_FeS_6_. After 4 months aging in air, the coercive field and Kerr rotation *θ*_K_ of Ta_3_FeS_6_ nanosheet remained unchanged, as shown in Figure 5c. The stability of Ta_3_FeS_6_ nanosheet was superior to recent reported 2D ferromagnetic materials, like CrI_3_, Cr_2_Ge_2_Te_6,_ and Fe_3_GeTe_2_.^[^ ^8^, ^12^, ^15^ ^]^ The high stability of 2D Ta_3_FeS_6_ may attribute to the larger electronegativity of sulfur and its chemical inertness to H_2_O and O_2_, suggesting its potential application in spintronic devices under the atmospheric environment.^[^ ^51^ ^]^

In summary, we have successfully prepared 2D Ta_3_FeS_6_ and investigated the ultra‐stable 2D ferromagnetism. Giant MOKE response was demonstrated in Ta_3_FeS_6_ nanosheet with Kerr rotation angle of 48 mrad and Curie temperature of ≈80 K. Most importantly, the Ta_3_FeS_6_ nanosheet exhibits outstanding air stability and its MOKE signal remains unchanged after 4 months of aging in air. We envision that the long‐life 2D ferromagnetic ordering of Ta_3_FeS_6_ will provide more opportunities for field‐effect modulation of magnetism and Curie temperature, magnetic doping engineering, and fabrication of magnetic tunneling junction working at the atmospheric environment.

## Experimental Section

##### Materials Preparation

2D Ta_3_FeS_6_ was prepared by mechanical exfoliation of Ta_3_FeS_6_ single crystal. Ta_3_FeS_6_ single crystals were synthesized by the I_2_‐assisted CVT method.^[^ ^23^, ^52^ ^]^ High‐pure Ta (676.7 mg, Alfa, 99.999%), Fe (100.2 mg, Alfa, 99.5%), and S (239.8 mg, Alfa, 99.999%) was mixed (molar ratio of 1:0.4:2) and then sealed under vacuum in a quartz tube with the addition of 200 mg I_2_ as the transport agent. Whereafter, the ampoule was placed in a two‐temperature zone horizontal tube furnace with the hot end at 1273 K and cold end at 1173 K in 10 h and kept for a week. Ta_3_FeS_6_ nanosheets were obtained by Au‐assisted as illustrated in Figure S1 (Supporting Information). First, single crystal Ta_3_FeS_6_ is placed on adhesive tape. Then Au film (200 nm) is evaporated onto the single crystal by thermal deposition. The Au atoms bond with the surface S atoms of the crystal. Next, the Au film was picked up by another tape along with some Ta_3_FeS_6_ flakes cohered on the surface. Finally, it's necessary to reduce the thickness of Ta_3_FeS_6_ flakes on Au film by mechanical exfoliation for several times.

##### Characterization

Ta_3_FeS_6_ crystals were characterized by an OM (BX53M, OLYMPUS), XRD (D2 PHASER, Bruker), a Raman spectrometer (Alpha 300RS+, WITec) equipped with a cryo console, and an AFM (Dimension Icon, Burke). The TEM, SAED, and EDS were performed in a field emission TEM (Tecnai G2 F20, FEI). The Au film was deposition in a high‐vacuum deposition system (Angstrom Engineering, Nexdep). MOKE measurements were carried out by using a home‐made microscopic polar MOKE system. The samples were placed in a superconducting magnet with a temperature range from 4.2 to 300 K and an out‐of‐plane magnetic field up to 5 T. The wavelength of laser source range from 500 to 900 nm with an excitation power of 3 µW. Incident light is normally incident on the sample surface through an aspheric lens. Mechanical chopper can change the incident light intensity and photoelastic modulator (PEM) can modulate the polarization of incident light. The combination of mechanical chopper and PEM can extract the MOKE signal of the sample. The detail of the optical setup is shown in Figure S3 (Supporting Information).

## Conflict of Interest

The authors declare no conflict of interest.

## Supporting information

Supporting Information
