# Supplementary material for: Air‐Stable 2D Intrinsic Ferromagnetic Ta3FeS6 with Four Months Durability
Source: Adv Sci (Weinh). 2020 Oct 13;7(22):2001722. doi: 10.1002/advs.202001722 (PMC7675181; doi:10.1002/advs.202001722)
Supplement: Supplementary file 1 — Supporting Information [file ADVS-7-2001722-s001.pdf]

© 2020 Wiley-VCH GmbH

ADVANCED SCIENCE
Open Access

Supporting Information

for *Adv. Sci.,* DOI: 10.1002/advs.202001722

Air-Stable 2D Intrinsic Ferromagnetic Ta_3FeS_6 with Four Months Durability

Jianwei Su, Mingshan Wang, Guiheng Liu, Huiqiao Li, Junbo Han, and Tianyou Zhai**

Supporting Information

Air-Stable Two-Dimensional Intrinsic Ferromagnetic Ta_3FeS_6 with Four Months Durability

Jianwei Su, Mingshan Wang, Guiheng Liu, Huiqiao Li, an, Tianyou Zhai**

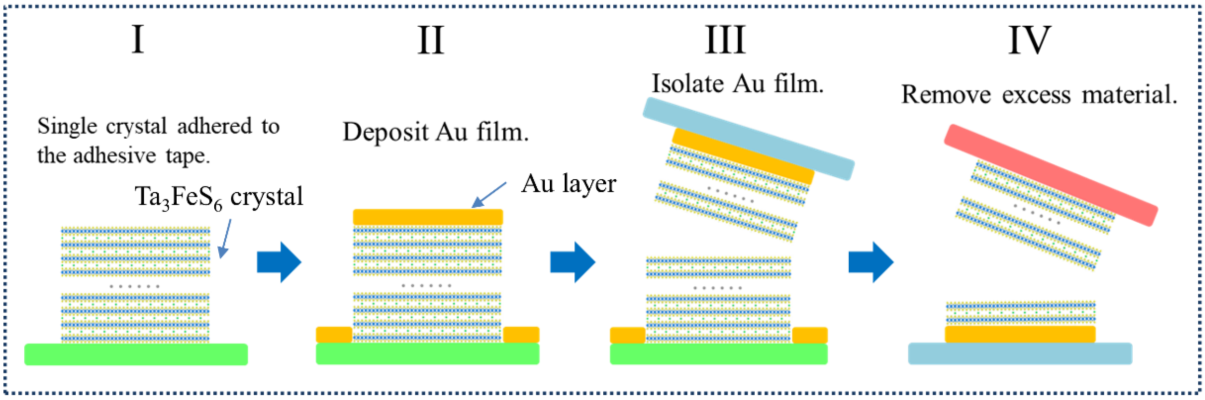

Figure S1. Schematic illustration of the Au-assisted Ta_3FeS_6 exfoliation process.

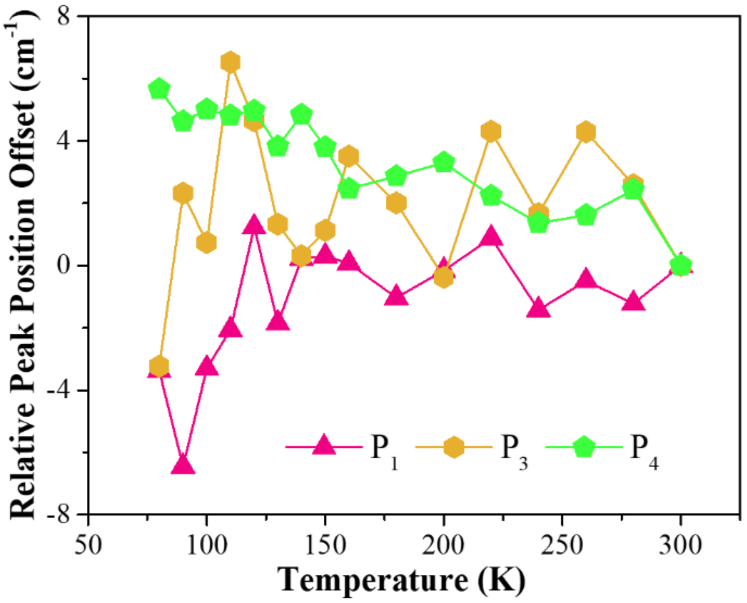

Figure S2. Temperature-dependent relative Raman peak position offset of P_1, P_3, and P_4.

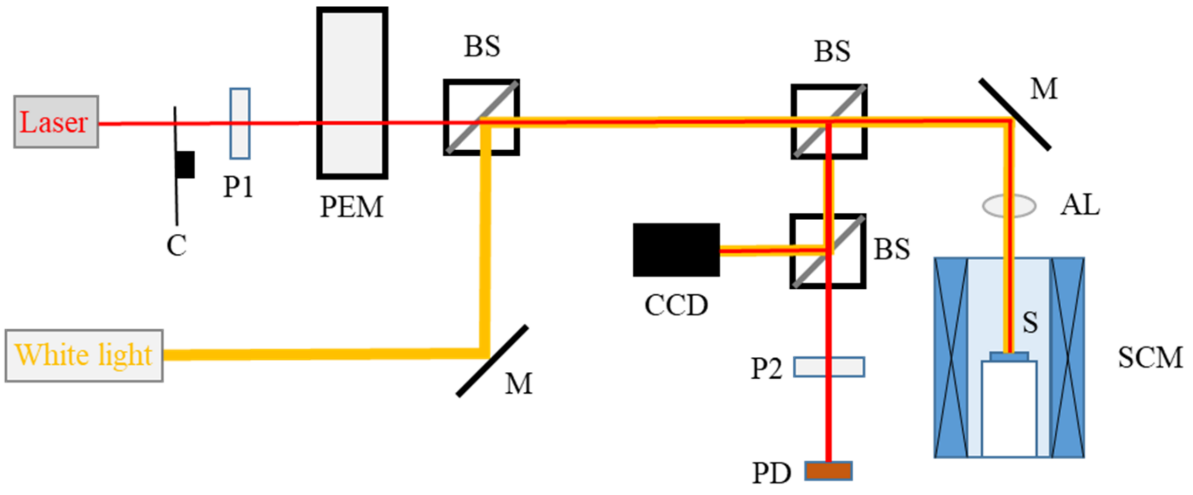

Figure S3. Schematic of the optical setup of polar MOKE measurement. C, chopper; P, polarizer; PEM, photoelastic modulator; BS, beamsplitter; PD, photodiode; M, silver mirror; AL, aspherical lens; SCM, superconducting magnet; S, sample.

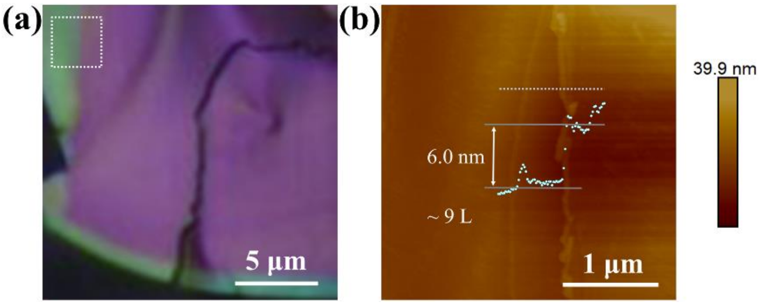

Figure S4. (a) OM image of Ta_3FeS_6 nanosheet obtained by Au-assisted exfoliation for MOKE measurement. (b) AFM characterization of Ta_3FeS_6 nanosheet acquired at the white square mark in (a).

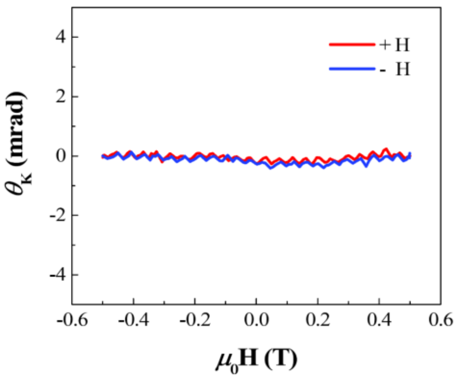

Figure S5. MOKE response of Au film at 10 K.

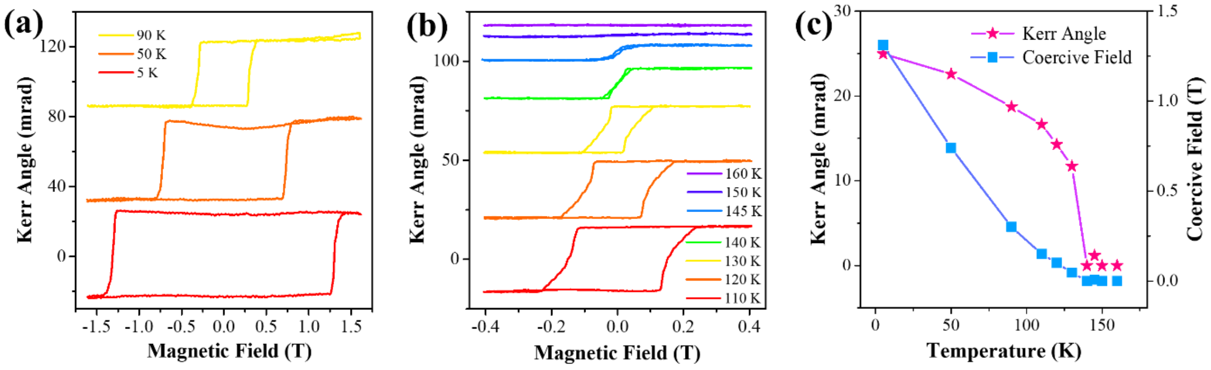

Figure S6. (a-b) Temperature-dependent polar MOKE measurement result of bulk Ta_3FeS_6. (c) Extractive Kerr angle and coercive field at different temperatures.

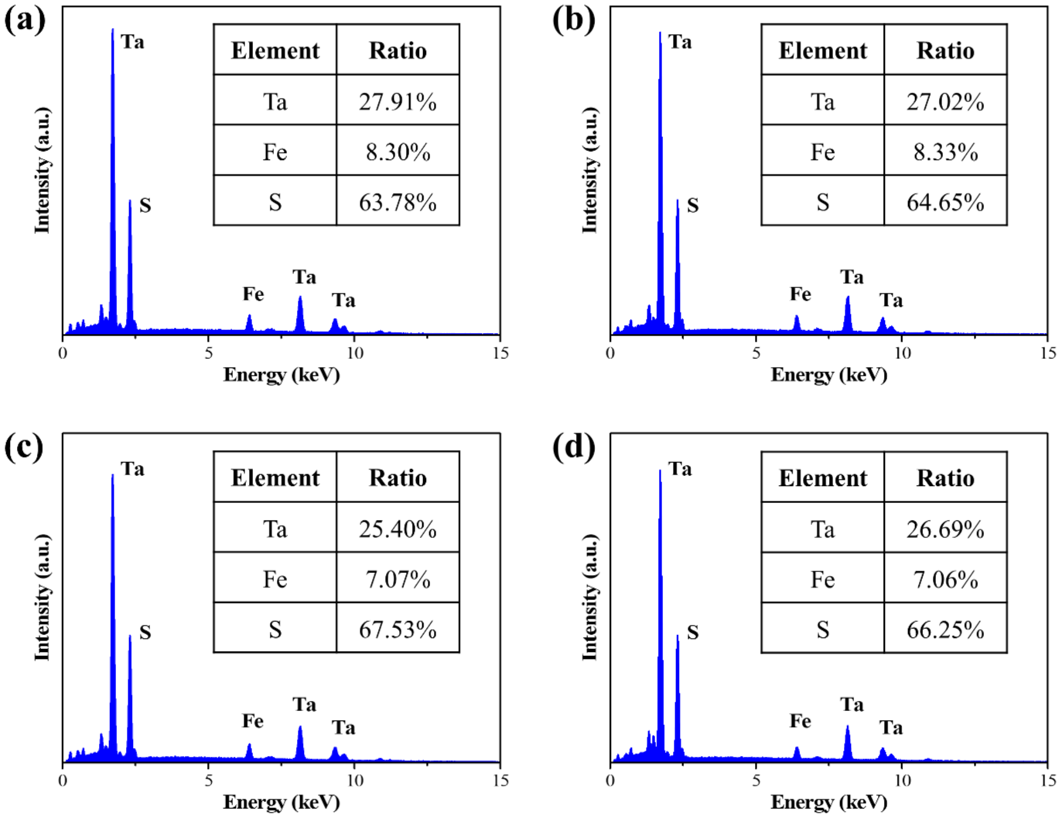

Figure S7. (a) EDS spectrum of single crystal Ta_3FeS_6. (b-d) EDS spectrum of exfoliated Ta_3FeS_6 crystal. The Fe content changed after mechanical exfoliation.

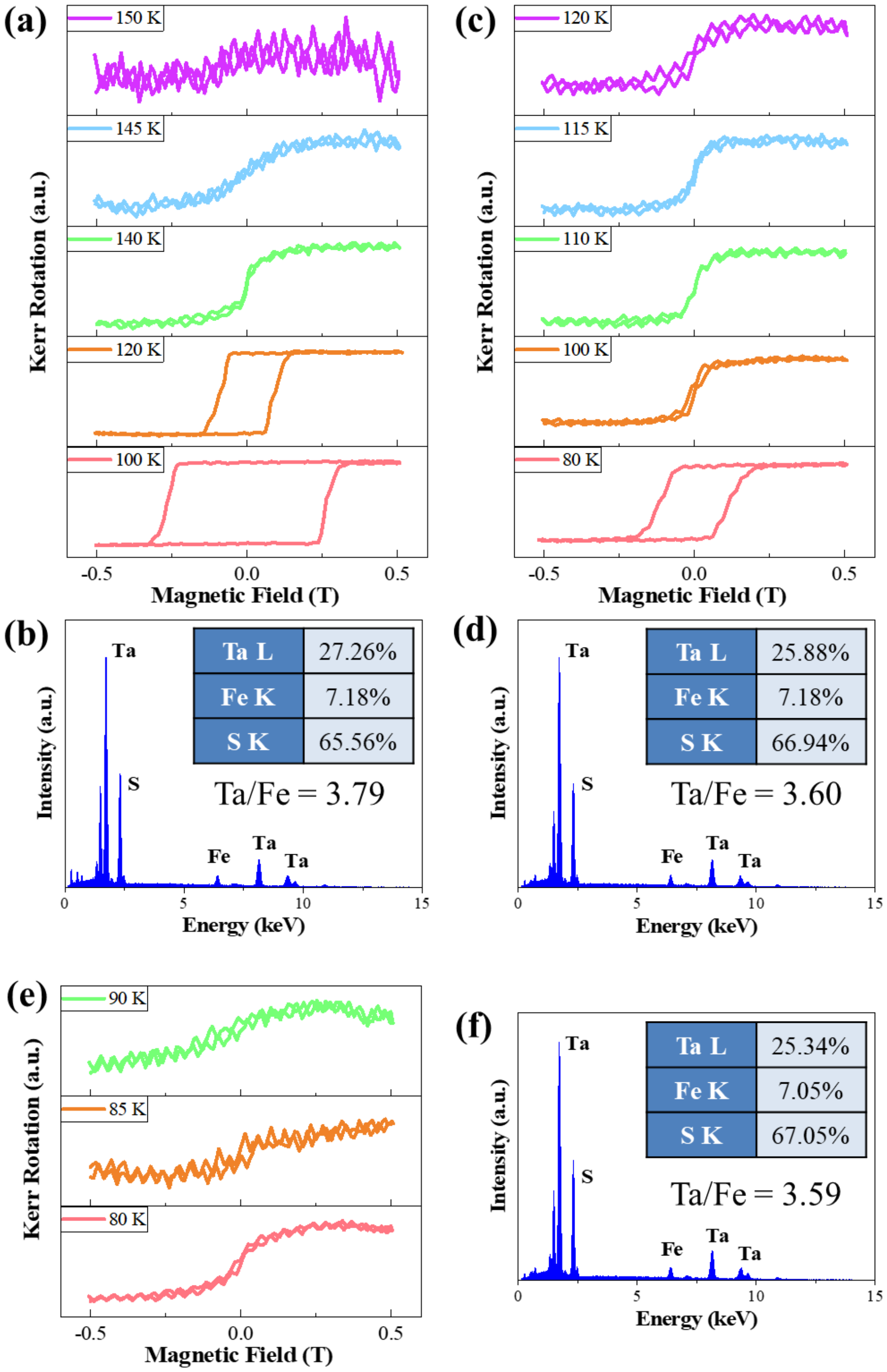

Figure S8. (a) MOKE measurement of Ta_3FeS_6 crystal with different Ta/Fe ratios.

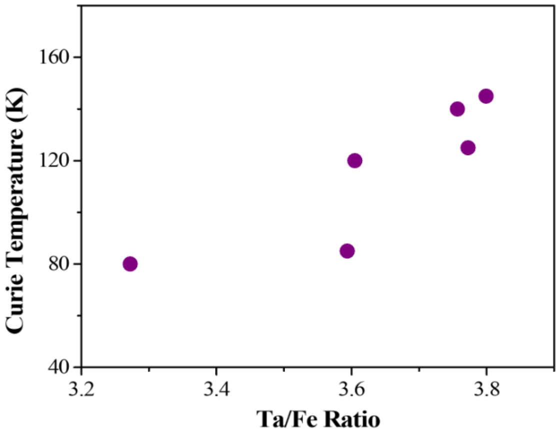

Figure S9. Curie temperature changing with different Ta/Fe ratios of Ta_3FeS_6.

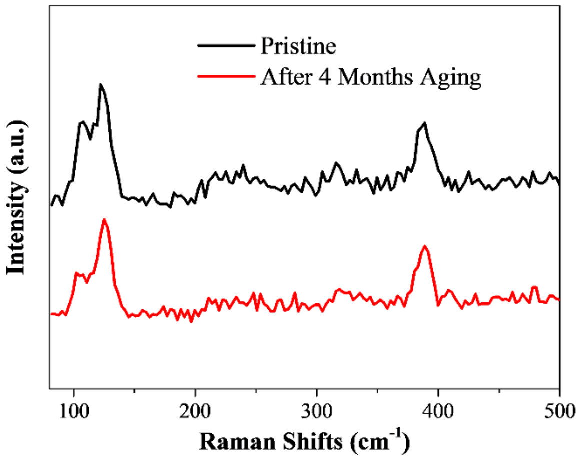

Figure S10. Raman spectra of Ta_3FeS_6 nanosheet before and after 4 months aging in the atmosphere.
